# Screening for postural orthostatic tachycardia syndrome using 24-hour electrocardiogram recording in patients with long coronavirus disease

**DOI:** 10.1016/j.hroo.2025.04.011

**Published:** 2025-05-08

**Authors:** David Hupin, Vincent Pichot, Magnus Bäck, Malin Nygren-Bonnier, Ulrika Reistam, Michael Runold, Judith Bruchfeld, Caroline Dupré, Antoine Da Costa, Cécile Romeyer, Frédéric Roche, Marcus Ståhlberg, Artur Fedorowski, Jannike Nickander

**Affiliations:** 1 Department of Medicine, Solna, Karolinska Institutet, Stockholm, Sweden; 2 Univ Jean Monnet, Department of Clinical and Exercise Physiology, University Hospital of Saint-Etienne, Mines Saint-Étienne, INSERM, U 1059, Saint-Étienne, France; 3 Department of Cardiology, Karolinska University Hospital, Stockholm, Sweden; 4 Division of Physiotherapy, Department of Neurobiology, Care Sciences and Society, Karolinska Institutet, Stockholm, Sweden; 5 Medical Unit Occupational Therapy and Physiotherapy, Women’s Health and Allied Health Professionals Theme, Karolinska University Hospital, Stockholm, Sweden; 6 Univ Jean Monnet, Department of Cardiology, University Hospital of Saint-Etienne, Mines Saint-Étienne, INSERM, U 1059, Saint-Étienne, France; 7 Department of Molecular Medicine and Surgery, Karolinska Institutet, Stockholm, Sweden; 8 Department of Clinical Physiology, Karolinska University Hospital, Stockholm, Sweden

**Keywords:** Autonomic dysfunction, Heart rate variability, Long COVID, Postural orthostatic tachycardia syndrome, 24-hour ECG recording

## Abstract

**Background:**

Cardiovascular autonomic dysfunction is a major complication in a large proportion of patients with long coronavirus disease (LC). As one of the most typical phenotypes of cardiovascular autonomic dysfunction, postural orthostatic tachycardia syndrome (POTS) is commonly observed as a sequelae of coronavirus disease infection.

**Objective:**

This study aimed to develop and test a 24-hour electrocardiogram (ECG) recording to direct the clinical suspicion toward the diagnosis of POTS.

**Methods:**

Consecutive patients referred to the Karolinska University Hospital in Stockholm from April 2021 to April 2022 were included. Patients with POTS were compared with patients with LC without POTS (verified by active standing tests) and control healthy subjects according to 3 specific analyses based on 24-hour ECG recording: (1) heart rate (HR) spikes of > 30 beats per minute, (2) awakening HR increase, and (3) HR variability (root mean square of successive difference). The control group consisted of healthy subjects from the database of the University Hospital of Saint-Etienne.

**Results:**

A total of 100 patients with LC (mean age, 42.54 ± 10.45 years; 92% women) and 100 healthy subjects (41.40 ± 7.21 years; 96% women) were included. LC POTS (n = 45) was associated with (1) a higher number of HR spikes/h (1.47 ± 0.84 vs 0.68 ± 0.50 and 0.40 ± 0.28/h; *P* < .01), (2) an abrupt and sustained increase in HR after awakening (*P* < .05), and (3) a reduction of HR variability: mean root mean square of successive difference of 34.90 ± 12.48 vs 30.47 ± 19.15 and 43.35 ± 21.10 ms (*P* < .01) compared with patients with LC without POTS (n = 55) and healthy subjects.

**Conclusion:**

A triple analysis of 24-hour ECG recordings could reveal a characteristic POTS signature in LC. More research in other populations is needed to draw any firm conclusions about its generalizability.

## Graphical abstract

**Figure:**
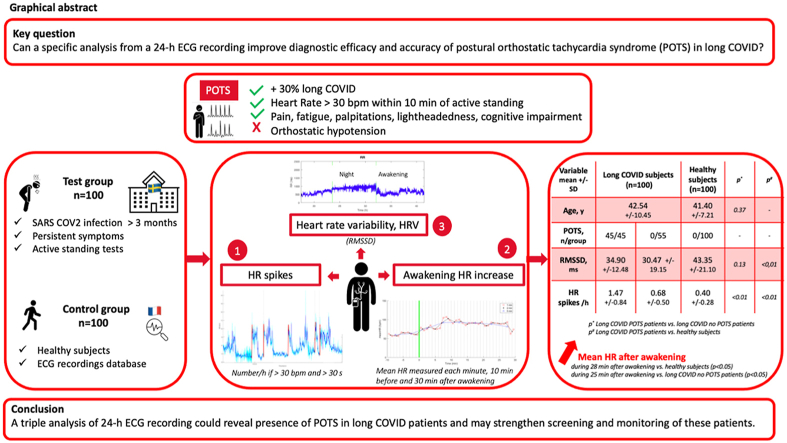

**Key Findings**

The study demonstrated that a triple analysis of 24-hour electrocardiogram (ECG) recordings:

▪ Identifies with a very high accuracy the presence of postural orthostatic tachycardia syndrome in long coronavirus disease patients, as confirmed by tilt test or active stand test.
▪ May help to raise awareness in healthcare professionals to systematically propose a 24-hour ECG to direct the clinical suspicion towards the diagnosis of postural orthostatic tachycardia syndrome.
▪ May substantially improve both diagnostic efficacy and accuracy.
▪ May allow enable early treatment access.

## Introduction

Although the coronavirus disease (COVID) 2019 (COVID-19) was officially declared over in May 2023, many patients continue to experience persistent symptoms, often referred to as long COVID (LC). These persistent and unexplained symptoms at least 3 months after COVID-19 infection include fatigue, pain, cognitive impairment (“brain fog”), breathlessness, palpitation, chest discomfort, sleep disturbance, and orthostatic intolerance.^1^, ^2^, ^3^ A substantial subset of patients with LC demonstrate signs of cardiovascular autonomic dysfunction with one of the most prevalent phenotypes of postural orthostatic tachycardia syndrome (POTS).^4^^,^^5^

The precise pathophysiology of POTS is not fully understood but several factors may contribute, including hyperadrenergic stimulation, hypovolemia, peripheral autonomic neuropathy, and autoimmunity.^6^ These mechanisms can occur together, and infections, surgery, pregnancy, psychological stress, and even vaccination have been identified as potential triggers.^6^, ^7^, ^8^, ^9^ COVID-19 infection has been associated with POTS-like symptoms regardless of the severity of the initial illness,^5^ and COVID-19 vaccination may also trigger POTS^4^^,^^10^^,^^11^ although the frequency is unknown.

POTS is diagnosed when a patient’s heart rate (HR) increases by more than 30 beats per minute (bpm) within 10 minutes of standing, without orthostatic hypotension, and with symptoms of orthostatic intolerance for at least 3 months.^6^^,^^12^, ^13^, ^14^, ^15^ If not correctly diagnosed and managed, POTS can lead to significant disability and impaired quality of life.^7^

The prepandemic prevalence of POTS was estimated to be 0.2%–1% of the population, mostly in young women. Since COVID-19, POTS-like symptoms have been observed in more than 10% of severe COVID-19 cases^16^ and a third of patients with LC.^4^^,^^5^ Although many patients recover within 1–2 years, delays in diagnosis and treatment are common owing to a long waiting time for tests, notably the head-up tilt test.

One useful diagnostic tool is a 24-hour electrocardiogram (ECG), which can detect arrhythmias, abnormal heart rhythm, palpitations, and HR reactivity abnormalities.^11^ A higher index of POTS detection from a simple and easily accessible 24-hour ECG in patients with LC and excessive tachycardia would improve overall diagnostic outcome and offer accurate therapeutic opportunities to prevent physical and psychological deterioration.^7^

## Methods

### Study population

The study included 100 patients with LC and 100 age- and sex-matched healthy controls examined before 2020. Consecutive patients with post-COVID were evaluated between April 2021 and April 2022 at Karolinska University Hospital in Stockholm, Sweden. All had a 24-hour ECG recording owing to persistent and unexplained cardiac symptoms (eg, palpitations, rapid heartbeats, and exercise/orthostatic intolerance) at least 3 months after COVID-19 infection.

COVID-19 diagnosis was confirmed by polymerase chain reaction testing. Patients with LC were categorized into 2 groups based on active standing or head-up tilt tests according to international consensus^15^: those with POTS (LC POTS+) and those without (LC POTS−). The control group was selected from a non-COVID database at Saint-Etienne University Hospital, France, from April 2018 to September 2019 with consecutive healthy subjects having no autonomic imbalance, cardiovascular diseases, or cardiovascular risk factors. This retrospective analysis of prospectively collected registry data was performed in accordance with the Declaration of Helsinki, and all participants have provided an informed written consent. The protocol was approved by the relevant ethics committee and by the institutional review boards for health pathway (1) for patients with COVID-19 persistent symptoms in France (ClinicalTrials.gov identifier: NCT05236478, NCT05787366) and (2) for healthy sportsmen in France (ClinicalTrials.gov identifier: NCT06024863).

### 24-hour ECG recording

The 2-lead 24-hour ECG recordings were analyzed using an ambulatory ECG device medilog recorder (Schiller, Baar, Switzerland) in the LC group and Vista Plus recorder (Novacor, Rueil-Malmaison, France) in the control group. ECG signals were visually inspected by 2 experts in the field (Jean-Claude Barthélémy^†^ and Vincent Pichot) using the medilog DARWIN2 software (Schiller, Baar, Switzerland) and HolterSoft Ultima software (Novacor, Rueil-Malmaison, France) to manually remove artifacts.

### HR spikes

Spikes were detected over the diurnal period of RR intervals derived from the 24-hour ECG recording. All subjects from the study wore an ambulatory ECG device with instructions to continue their usual activities. This calculation aims to detect rapid HR increases of > 30 bpm and maintained for a duration of > 30 seconds, as described in POTS populations during a standing position.^6^^,^^11^ These RR variations are different than those after sleep apnea and periodic leg movements in sleep and wakefulness in populations with sleep disorders.^17^^,^^18^ The RR signal was first transformed into an instantaneous HR signal, HR_i_ (bpm) = 60,000/RR_i_ (ms), and resampled at a fixed frequency of 1 Hz, then smoothed with a low-pass filter to remove fast HR oscillations lasting < 10 seconds (Butterworth IIR filter order 12 with a cutoff frequency of 0.1 Hz). From the signal obtained, the algorithm then searches for all sequences meeting the following 3 criteria: (1) rapid rise (< 30 seconds) in HR of > 30 bpm, (2) increase in HR maintained for > 30 seconds, and (3) lasting < 5 minutes. The result was then reduced to a number of spikes/h.

### Awakening HR increase

The awakening was analyzed with the 24-hour ECG recording. It is asked of each subject to keep the 24-hour ECG recording 1 hour at least after awakening and to report physical activity periods. In addition, HR variability (HRV) analysis was used to distinguish between changes in HR owing to physical activity and other factors such as stress or rest. For each subject, the night period was manually detected using the sleep/wake times entered by the subjects and checked using times on the 24-hour ECG recording. The precise moment of the alarm clock was refined by taking the awakening tachycardia visible in all subjects. The patients who had missing awakening time data or insufficient alarm clock analysis time were excluded from the analysis. The change in awakening HR was measured by calculating the difference between the mean HR measured each minute, each 2 minutes and each 5 minutes, 10 minutes before and 30 minutes after awakening, according to the sleep diary completed by patients.

### HRV

The RR series arising from the processed 24-hour ECG signals was used for a time- and frequency-domain HRV analysis^19^ with an HRV analysis software.^20^ This software allows HRV analysis for the entire recording period using the 24-hour ECG recording or day and night separately. Interpretation of HRV indices remains especially debated in long-term recordings because major determinants of HRV such as environmental factors, physical activity, and sleep duration vary significantly over time.^20^ For these reasons, only nocturnal HRV was analyzed to allow comparison between subjects. For each subject, the night period was manually detected using the sleep/wake times entered by the subjects and the bradycardia/tachycardia resulting from the sleep/wake times visible on the 24-hour RR signal. After interpolating isolated extrasystoles or artifacts and excluding excessively artifacted periods from the analyses, the autonomic nervous system activity was assessed from the time- and frequency-domain HRV parameters. Time-domain measurements included the average RR interval (in ms), the standard deviation of the interbeat interval NN (SDNN, in ms), and the root mean square of successive differences (RMSSDs) between normal heartbeats (RMSSD, in ms). Frequency-domain parameters included the low frequency (LF) (0.04–0.15 Hz) and high frequency (HF) bands (0.15–0.40 Hz). The ratio of LF to HF bands was also calculated.^21^ Briefly, the RMSSD and HF are regarded as indicators of parasympathetic influence on the HR, whereas the SDNN and LF component have a complex physiology that integrates both the sympathetic and parasympathetic components, and the LF-to-HF ratio has been proposed to approximate sympathovagal balance.^22^^,^^23^

### Statistical analysis

Normal distribution of continuous variables was tested by the Kolmogorov–Smirnov test. Continuous variables were expressed as means ± standard deviations. Differences among the 3 main groups were analyzed for statistical significance using the unpaired *t* test, Mann–Whitney U test, or χ^2^ analysis, as appropriate.

The classification accuracy of the measure of HR spikes, then awakening mean RR kinetics, and finally HRV associated with detecting POTS is summarized in a 2 × 2 contingency table, which shows a dichotomous classification, with positive (POTS) and negative outcomes (no POTS). The diagnostic performance of this triple analysis of 24-hour ECG recordings was assessed by sensitivity (se), specificity (sp), and positive (LR+) and negative likelihood ratios (LR−), which are prevalence independent. Nevertheless, each of these indicators only partially assesses the diagnostic performance because they only focus on positive (POTS) or negative cases (no POTS). The receiver operating characteristic (ROC) curve has been produced to encompass both positive and negative cases so that it can reflect the global ability of the triple analysis to discriminate between the diagnostic groups. The ROC curve (adjusted for age and sex) was produced by plotting se (true positive rate) on the y-axis against 1-sp (false positive rate) on the x-axis for the HR spikes and HRV values (RMSSD). The area under the ROC curve (AUC) is a global measure of the ability of the test to discriminate whether POTS was present or not present in the function of both variables of interest. The Youden index is a commonly used approach when selecting a cutoff point to give equal weight to the importance of se and sp by choosing the point nearest to the top-leftmost corner of the ROC curve.^24^ In addition to J, the optimal cut point has been determined from the point on the ROC curve closest to (0,1) and above all a generalized J, which considers the prevalence.^25^ These thresholds were best illustrated using the conventional 2 × 2 table. Se and sp, LR+ and LR−, and AUC are reported as markers of accuracy. A linear mixed model was used to compare mean HR at the different time points surrounding the awakening until 30 minutes. Time, awakening, and time × awakening interaction were specified as fixed effects, whereas subjects were defined as a random effect. Several models were tested according to different combinations of the included fixed effects. The effect of each predictor was assessed using a Wald test. The model that provided the lowest Bayesian information criterion was retained for the results. Simple linear regressions were also performed to compare mean HR values at different time points among the 3 main groups. Regression lines were calculated for each group (y = ax + b). All analyses were performed using R software (version 4.1.2), where *P* < .05 was considered statistically significant.

## Results

**Figure 1:**
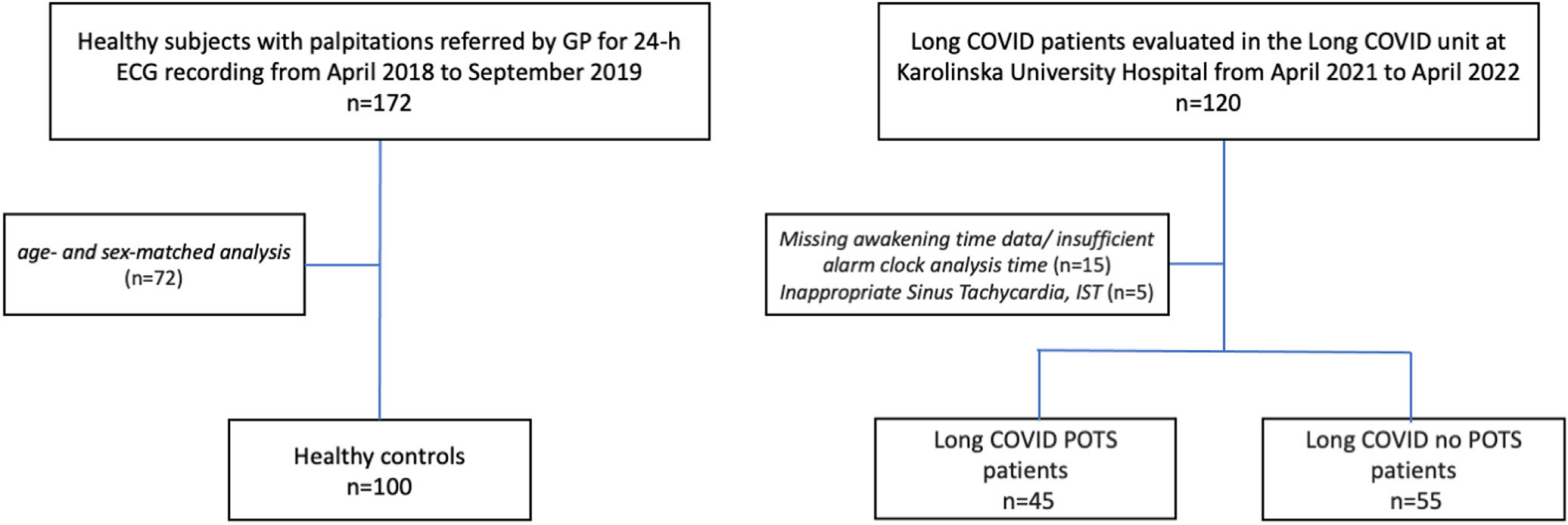
Flowchart. COVID = coronavirus disease; ECG = electrocardiogram; GP = general practitioner; POTS = postural orthostatic tachycardia syndrome.

The study population included 100 consecutive patients with LC (92% women) and 100 consecutive healthy subjects (96% women) (Figure 1).

Mean age was 42.54 ± 10.45 years, ranging from 19 to 62 years for patients with LC, and 41.40 ± 7.21 years, ranging from 25 to 55 years for healthy subjects (*P* = .37) (Table 1).

**Table 1:** Descriptive of patients’ characteristics

Variables, mean ± SD	Subjects with long COVID (n = 100)		Healthy subjects (n = 100)	*P* value∗	*P* value†
Age, y	42.54 ± 10.45		41.40 ± 7.21	.37	
Female, n (%)	92 (92)		96 (96)	.24	
POTS, n/group	45/45	0/55	0/100		
ECG duration, h	24.67 ± 6.51	24.22 ± 7.28	22.10 ± 1.48	.37	.08
RR, ms	722.46 ± 82.22	801.21 ± 103.62	799.04 ± 120.92	<.01	<.01
RMSSD, ms	34.90 ± 12.48	30.47 ± 19.15‡	43.35 ± 21.10	.13	<.01
SDNN, ms	90.61 ± 23.73	86.97 ± 35.24‡	97.33 ± 29.90	.47	.04
LF-to-HF ratio	3.78 ± 1.55	4.17 ± 1.88‡	2.84 ± 1.43	.29	.01
HR spikes/h	1.47 ± 0.84	0.68 ± 0.50	0.40 ± 0.28	<.01	<.01
HR spikes, n/24 h	15 ± 9.4	6 ± 4.2	4 ± 2.5	<.01	<.01

COVID = coronavirus disease; ECG = electrocardiogram; HF = high frequency; HR = heart rate; LF = low frequency; POTS = postural orthostatic tachycardia syndrome; RMSSD = root mean square of successive difference; SD = standard deviation; SDNN = standard deviation of the interbeat interval NN.

∗ Patients with long COVID with POTS vs patients with long COVID without POTS.

† Patients with long COVID with POTS vs healthy subjects.

‡ *P* < .01 between patients with long COVID without POTS and healthy subjects.

**Figure 2:**
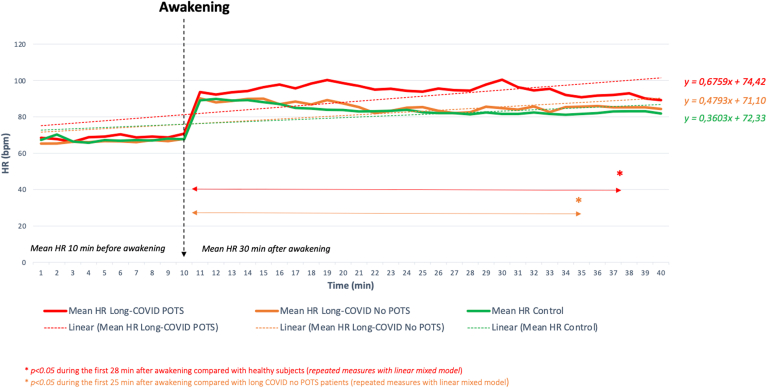
Analysis of the kinetics of the mean HR surrounding the awakening of (1) patients with long COVID with POTS, (2) patients with long COVID without POTS, and (3) healthy control subjects. COVID = coronavirus disease; HR = heart rate; POTS = postural orthostatic tachycardia syndrome.

LC POTS+ (n = 45; 45%) were associated with the following findings: (1) a higher number of HR spikes/h compared with healthy subjects (1.47 ± 0.84 vs 0.40 ± 0.28/h; *P* < .01) and LC POTS− (1.47 ± 0.84 vs 0.68 ± 0.50; *P* < .01), (2) a more abrupt and sustained increase in mean HR during the first 28 minutes after awakening compared with healthy subjects (*P* < .05) and the first 25 minutes after awakening compared with LC POTS− (*P* < .05) (Figure 2), and (3) a greater decrease in most HRV parameters than healthy subjects (mean SDNN, 90.61 ± 23.73 vs 97.33 ± 29.90 ms; *P* = .04). The most reduced components for LC POTS+ were those related to the parasympathetic tone: mean RMSSD of 34.90 ± 12.48 vs 43.35 ± 21.10 ms in healthy subjects (*P* = .04) (Supplemental Figures 1–7).

The measures of se and sp with accuracy to detect POTS from each of these parameters are presented in Supplemental Table 1. The ROC curves have been used to determine appropriate cutoffs, affecting the se and sp of the test (Supplemental Figures 8 and 9). The threshold selected for HR spikes was 0.9 spikes/h (se = 0.82 and 1-sp = 0.09) then for RMSSD was 35 ms (se = 0.52 and 1-sp = 0.43). The significant data from the linear mixed model were used for HR after awakening. When these 3 parameters (HR spikes, HR after awakening, and HRV [RMSSD]) were associated, se = 91.11% (91.11%), sp = 99% (96.36%), LR+ = 25.05 (91.11), and LR− = 0.09 (0.09) between LC POTS+ and healthy subjects (between LC POTS+ and LC POTS−).

## Discussion

Our study demonstrated that a triple analysis of 24-hour ECG recordings, including (1) assessing HR spikes, (2) an HR increase after awakening, and (3) a reduced HRV, accurately identifies POTS-like in patients with LC as confirmed by tilt test or active stand test.

LC POTS+ often presents with sinus tachycardia, orthostatic intolerance, and cardiovascular dysautonomia,^4^^,^^11^ with HRV serving as a key noninvasive biomarker for autonomic dysfunction in subjects with post-COVID-19.^26^ Several studies have reported reduced HRV in LC POTS+ compared with LC POTS− or healthy.^26^, ^27^, ^28^, ^29^, ^30^, ^31^ HRV, particularly RMSSD, is an important indicator of parasympathetic tone and a marker for cardiovascular dysautonomia,^26^ with reduced RMSSD observed in patients with LC POTS+ compared with healthy controls. Our study aligns with previous findings of lower HRV in LC POTS+,^32^ reflecting cardiac autonomic imbalance, including increased sympathetic activity and decreased parasympathetic tone.^5^^,^^29^ Although some studies suggest an increased parasympathetic tone in patients with LC,^33^^,^^34^ our results point to a predominant sympathetic overactivity from HRV analysis,^6^ contributing to the characteristic POTS symptoms such as tachycardia, fatigue, neurocognitive disorder, and altered sleep structure.^6^^,^^11^^,^^35^^,^^36^ In addition, the autonomic nervous system modulates the inflammatory response, and reduced HRV is associated with higher inflammatory response through the cholinergic anti-inflammatory pathway,^37^, ^38^, ^39^ suggesting a need for tailored exercise rehabilitation to address both autonomic and inflammatory dysregulation.

Currently, POTS diagnosis relies on specialized tests such as the tilt test,^4^ but these are often inaccessible owing to long wait times.^2^ Even if an active stand test is correctly performed, its results may be inconsistent or inconclusive because POTS may fluctuate over time and performing an additional and easily available examination, a 24-hour ECG recording, may substantially improve both diagnostic efficacy and accuracy.

Among the strengths of the study, the ROC curve graphically displayed the trade-off between se and sp and was useful in assigning the best cutoffs for clinical use. The AUC also provided a useful parameter for comparing test performance.^24^^,^^25^ The threshold selected for HR spikes was robust: 0.9 spikes/h with se = 0.822, 1-sp = 0.098, and AUC of > 0.95. The increase kinetics for HR after awakening was relevant because it was from linear mixed models and finally the third parameter, HRV with RMSSD, had a modest accuracy: 35 ms with se = 0.52, 1-sp = 0.43, and AUC of > 0.50. From ROC curves of SDNN and LF-to-HF ratio, the low accuracy of detecting POTS did not justify considering these HRV parameters as relevant. Thus, these 3 thresholds (HR spikes ≥ 0.9/h, immediate and sustained increase of HR after awakening during 25 minutes at least, and RMSSD ≤ 35 ms) were associated with a robust accuracy: high se > 90%, very high sp > 98%, very high LR+ > 10, and very high LR− < 0.1.

Some important study limitations should be mentioned. A 24-hour ECG recording does not measure arterial blood pressure compared with active standing test/head-up tilt testing, which can diagnose orthostatic hypotension.^40^ In addition, there were no standing tests or head-up tilt testing performed among the healthy subjects, which may explain the uncertain prevalence among healthy subjects in the control group. The nonrandomized study design and the relatively small sample size were major limitations of this study. Se/sp analyses of the main thresholds from the 24-hour ECG recording and external validation of the model with other cohorts could help improve diagnostic accuracy. The study population was strongly dominated by female sex, which limits the generalizability of study observations for male subjects. The control group was not selected from the same time period as patients with LC, which could introduce bias. In addition, the separate populations of Swedish patients with LC and healthy French subjects may have inherent differences, and thus, the comparisons between these populations should be interpreted with caution.

However, this is the first study that examined the use of a 24-hour ECG recording to detect POTS characteristics in patients with LC. In addition, 24-hour ECG recording offers a unique opportunity for precision tachycardia phenotyping, which is usually lacking in similar studies.^41^ Before the study, only reduced HRV parameters were found to be a simple, noninvasive biomarker for autonomic dysfunction in subjects with post-COVID-19^26^ but less specific for the diagnosis of POTS. This study focuses on the clinical relevance of simultaneously assessing 3 physiological parameters in patients with LC POTS. It is not intended to establish or validate a diagnostic scoring system. The high concordance observed in triple-positive patients supports their potential diagnostic value. Until more evidence emerges on whether physiopathology in LC POTS+ is different from non–COVID-19-related POTS, the next step will aim to develop and validate a diagnostic algorithm, including the definition of thresholds (eg, 2/3 vs 3/3 criteria), weighting strategies, and ROC analyses.

## Conclusion

A triple analysis of 24-hour ECG recordings in LC POTS+ revealed an increased number of HR spikes, prolonged increase in HR after awakening, and significantly reduced HRV. This novel analysis may be introduced in addition to the clinic for accelerating screening and therapy monitoring in LC-related cardiovascular autonomic dysfunction.

## Disclosures

The authors have no conflicts to disclose.
