# Supplementary material for: Screening for postural orthostatic tachycardia syndrome using 24-hour electrocardiogram recording in patients with long coronavirus disease
Source: Heart Rhythm O2. 2025 May 8;6(7):949–55. doi: 10.1016/j.hroo.2025.04.011 (PMC12302153; doi:10.1016/j.hroo.2025.04.011)
Supplement: Supplementary Figures 1 to 7 [file mmc1.pdf]

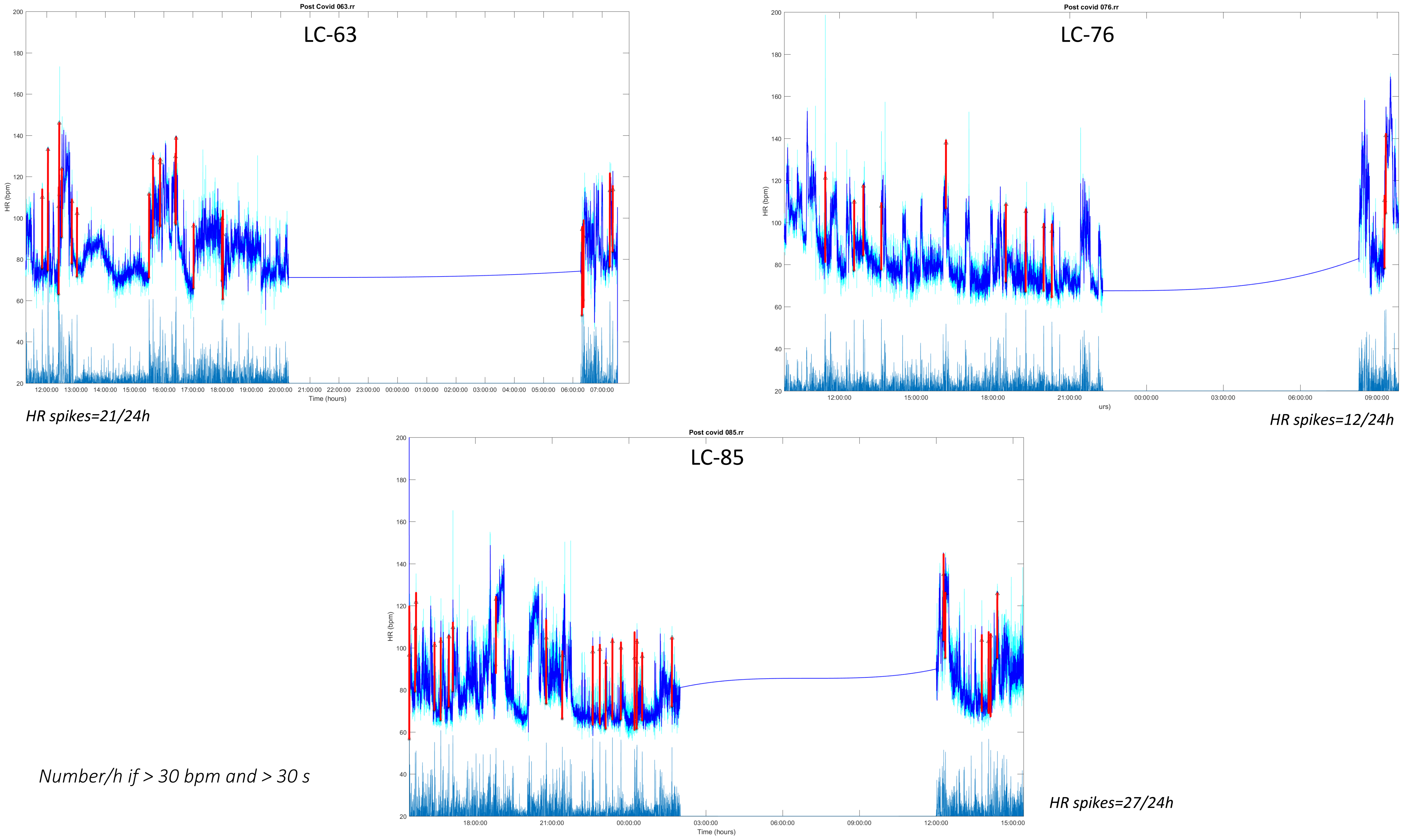

Supplementary Figure 1. Detection of heart rate spikes in long COVID POTS patients over the diurnal period of RR intervals derived from 24h ECG recordings

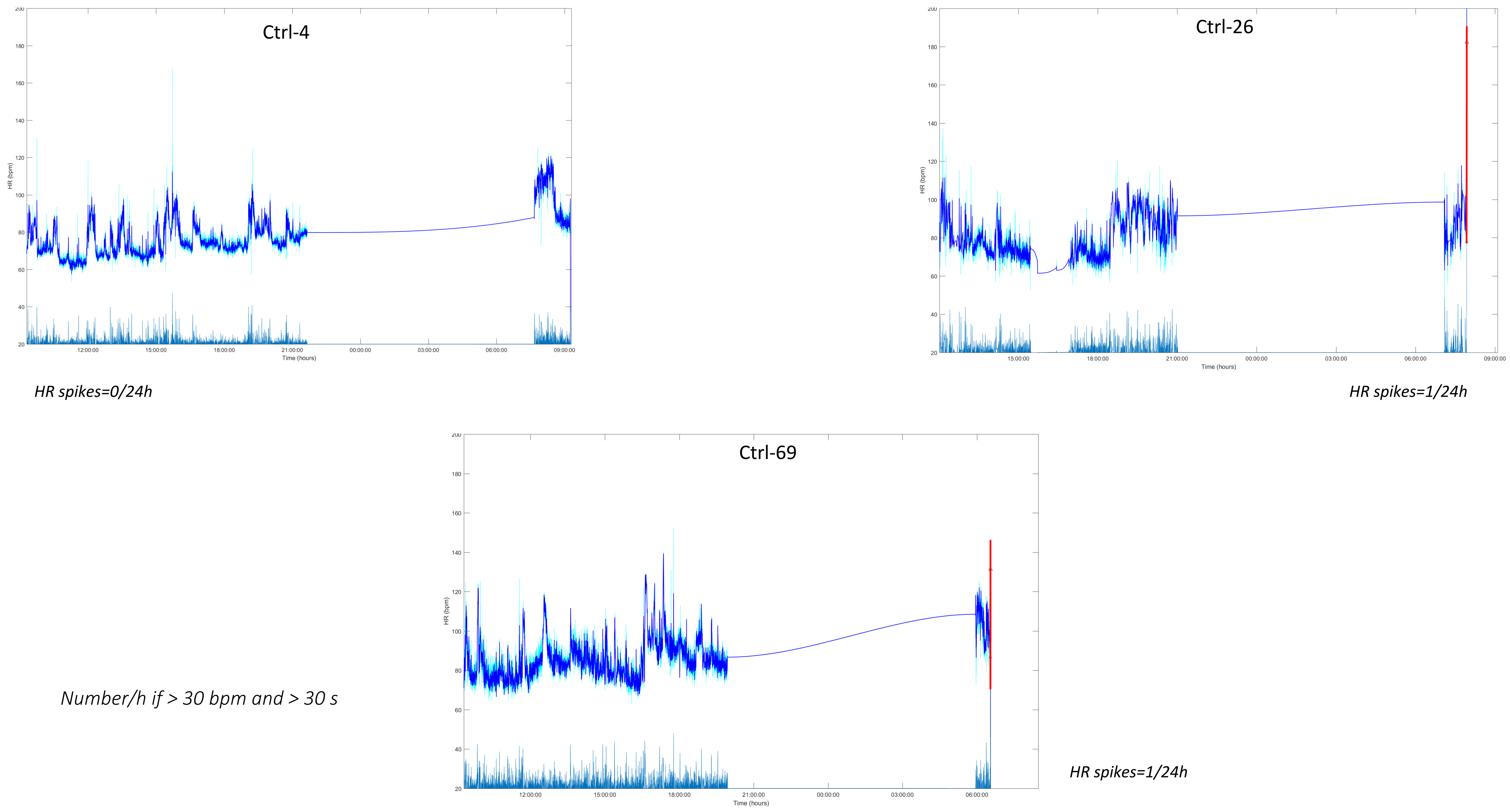

Supplementary Figure 2. Detection of heart rate spikes in long COVID no POTS patients over the diurnal period of RR intervals derived from 24h ECG recordings

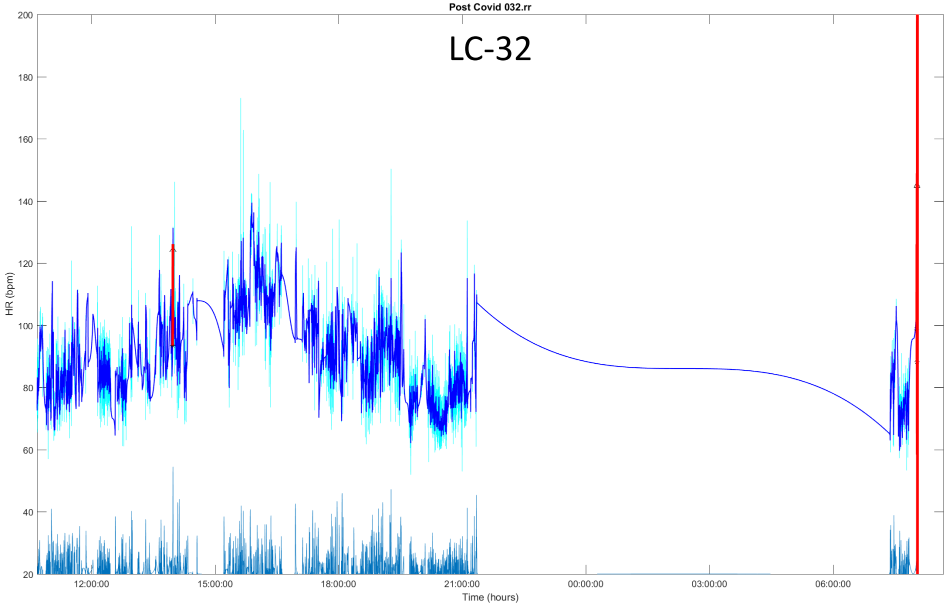

HR spikes=5/24h

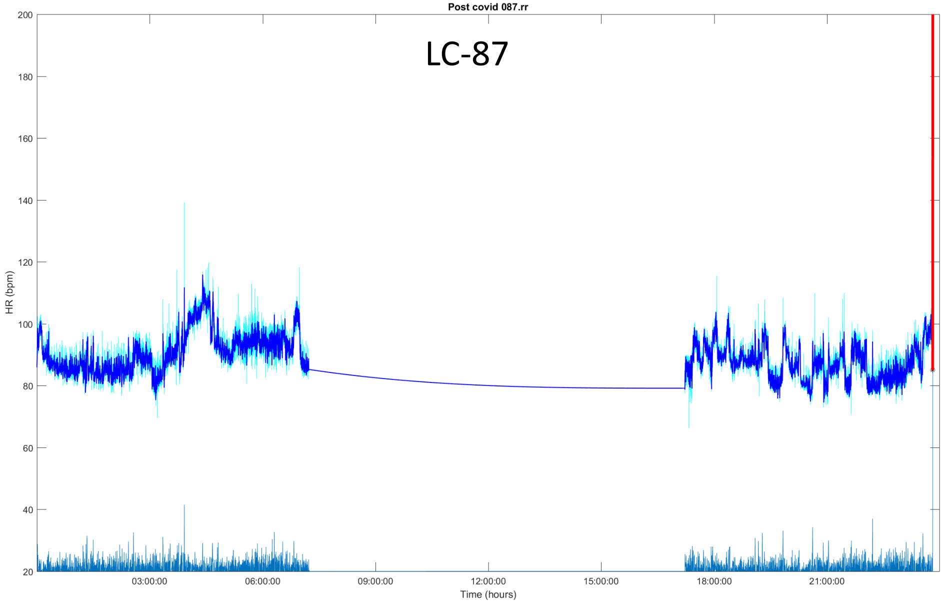

HR spikes=2/24h

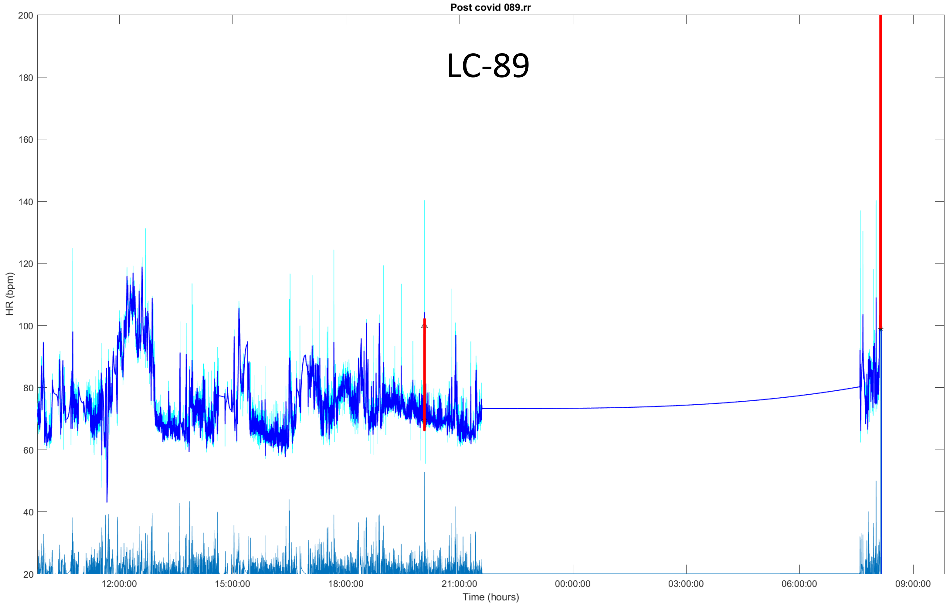

Number/h if > 30 bpm and > 30 s

HR spikes=2/24h

Supplementary Figure 3. Detection of heart rate spikes in healthy controls subjects over the diurnal period of RR intervals derived from 24h ECG recordings

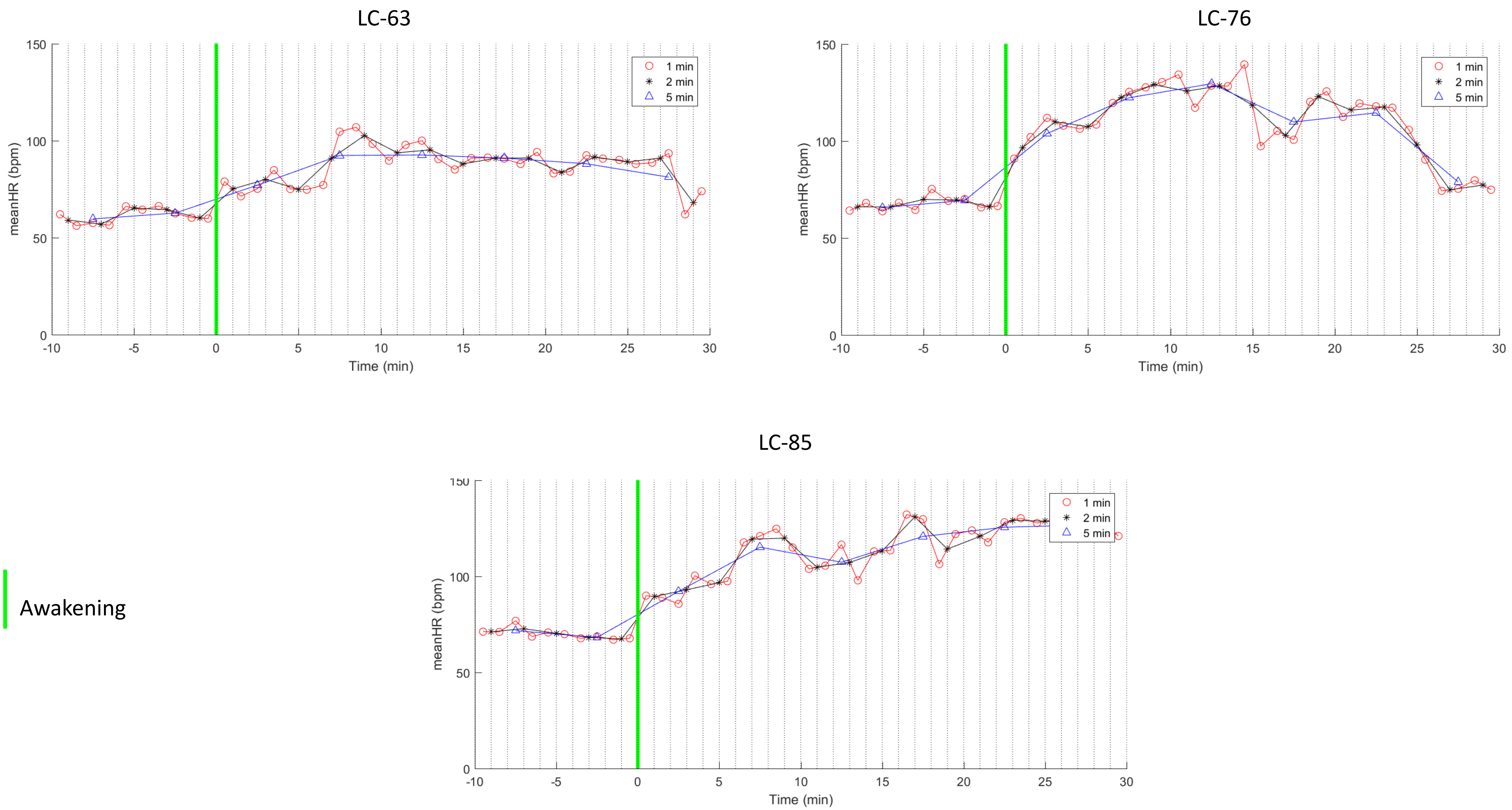

Supplementary Figure 4. Analysis of the mean HR surrounding the awakening of long COVID POTS patients

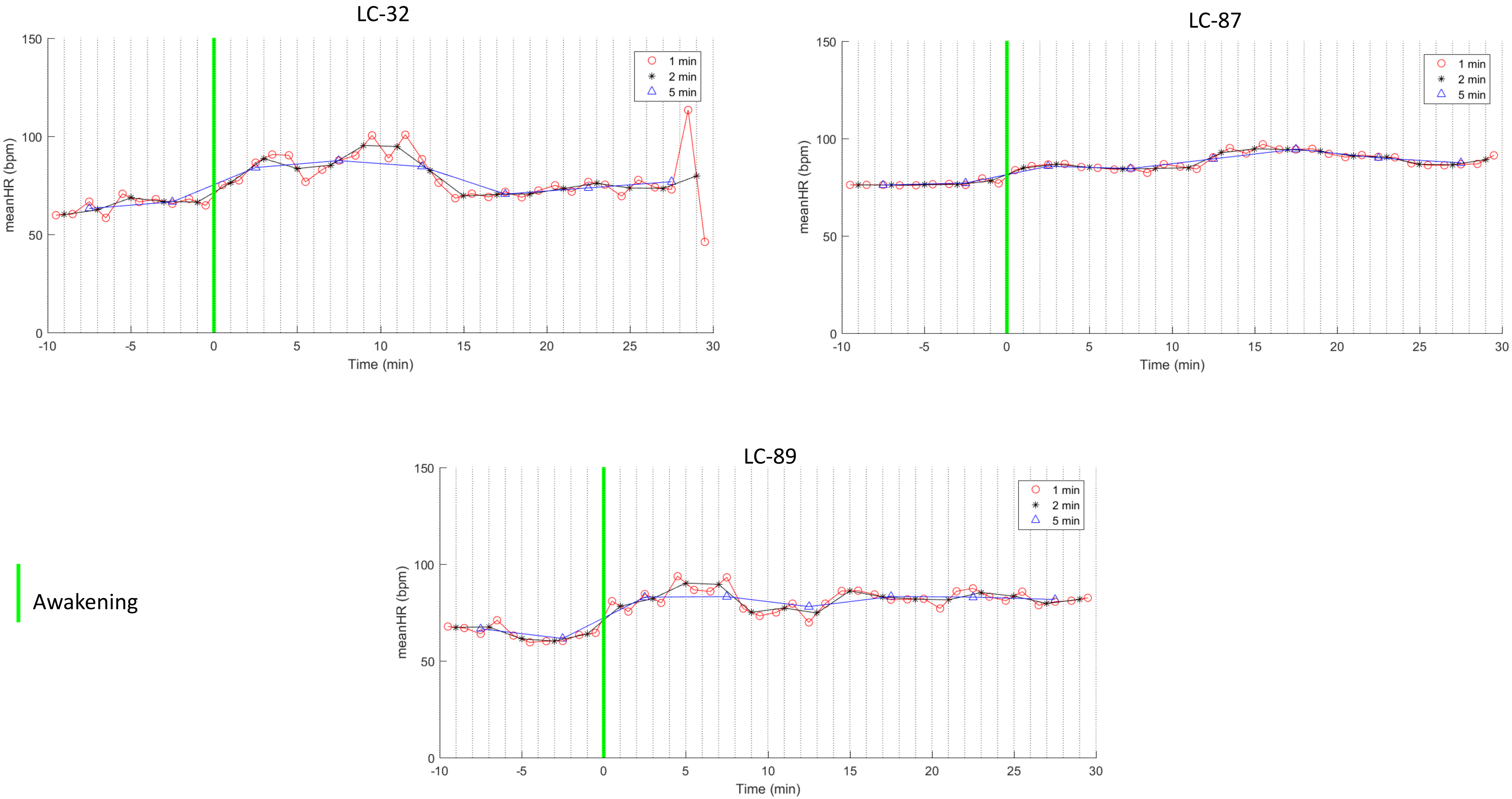

Supplementary Figure 5. Analysis of the mean HR surrounding the awakening of long COVID no POTS patients

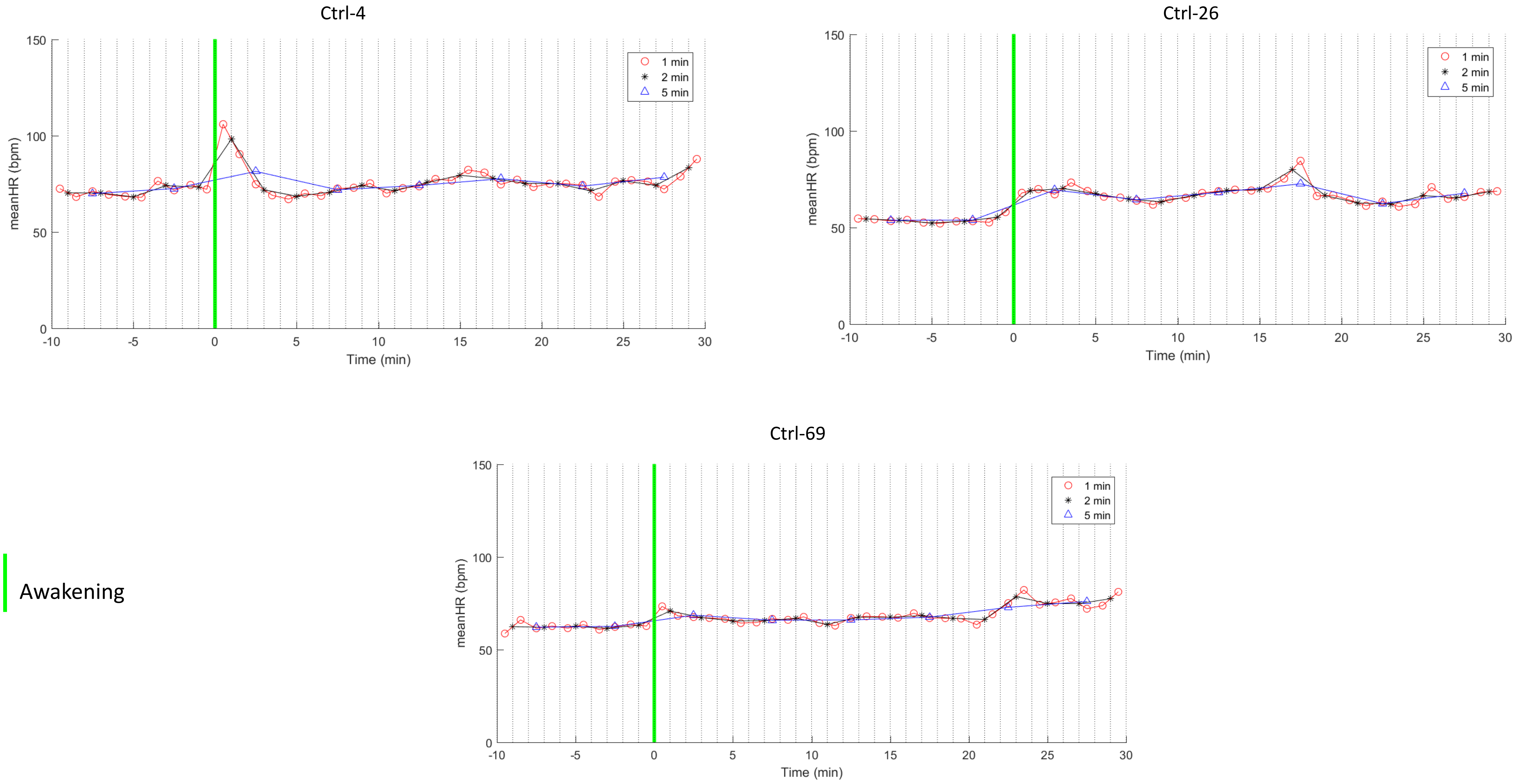

Supplementary Figure 6. Analysis of the mean HR surrounding the awakening of healthy controls subjects

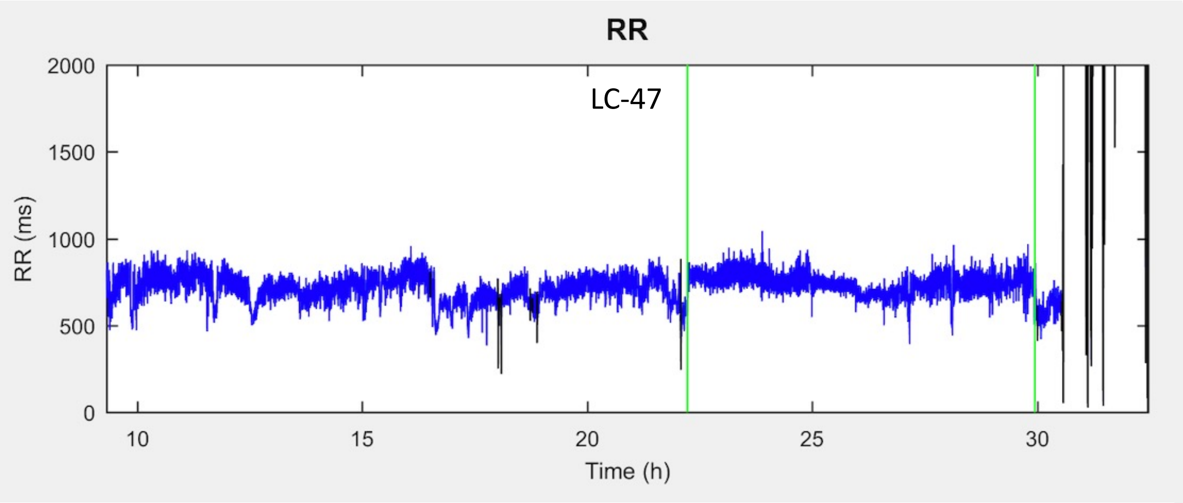

SDNN= 58 ms, RMSSD=17 ms, LF/HF= 6.6

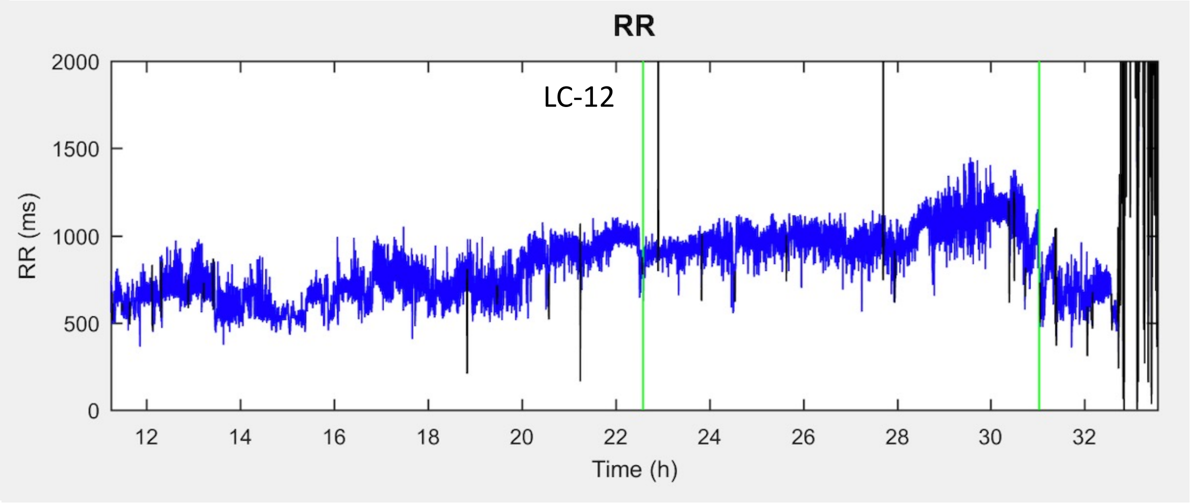

SDNN= 100 ms, RMSSD=30ms, LF/HF=4.8

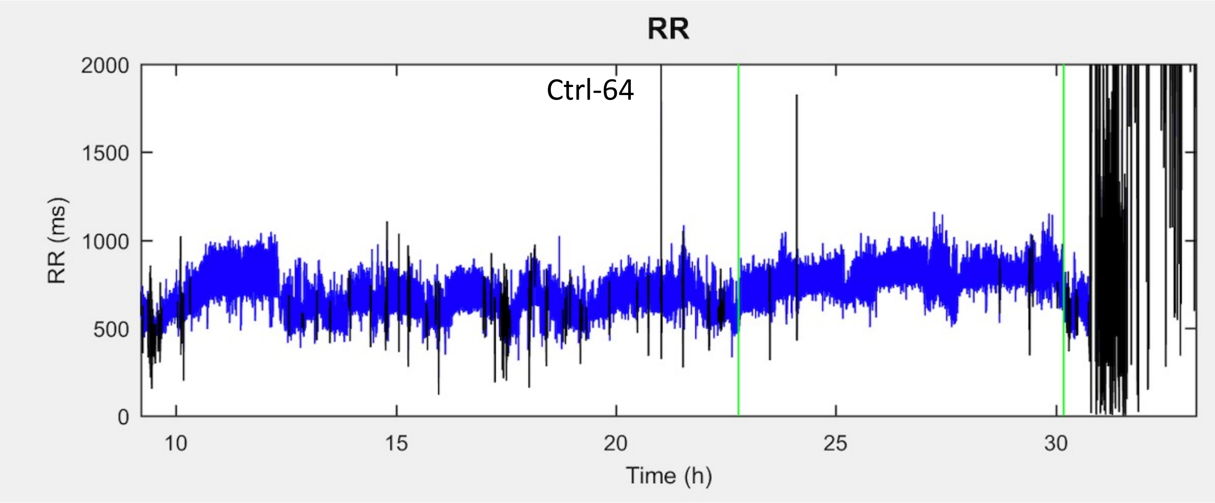

SDNN= 82 ms, RMSSD=52ms, LF/HF=1.8

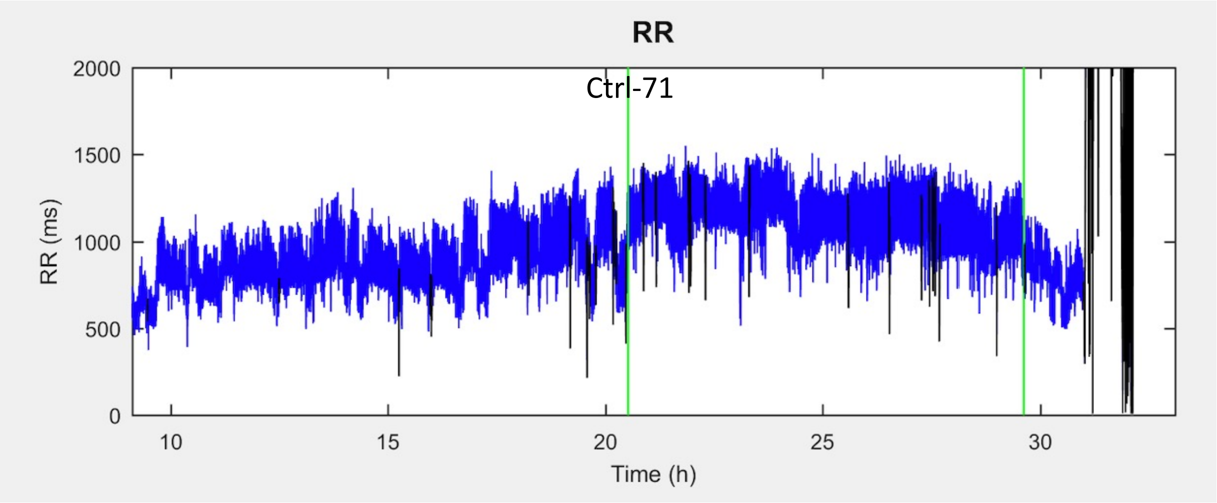

SDNN= 143 ms, RMSSD=97ms, LF/HF=1.2

Supplementary Figure 7. Heart rate variability analysis over the nocturnal period of RR intervals derived from 24h ECG recordings
