# Supplementary material for: Screening for postural orthostatic tachycardia syndrome using 24-hour electrocardiogram recording in patients with long coronavirus disease
Source: Heart Rhythm O2. 2025 May 8;6(7):949–55. doi: 10.1016/j.hroo.2025.04.011 (PMC12302153; doi:10.1016/j.hroo.2025.04.011)
Supplement: Supplementary Figures 8 to 9 [file mmc2.pdf]

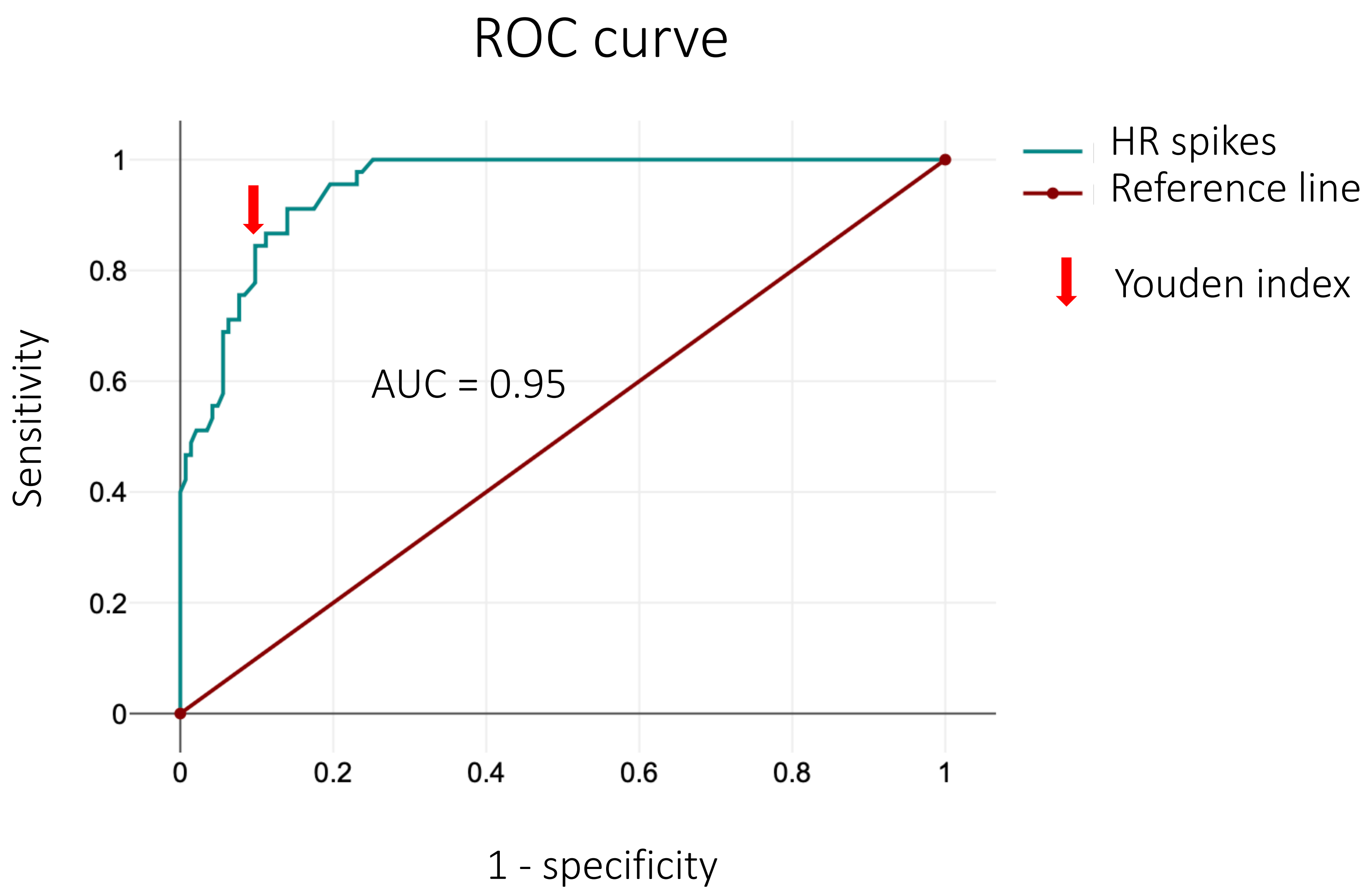

Supplementary Figure 8. Heart rate spikes relevance characteristic curve for postural orthostatic tachycardia syndrome using data from derivation set. AUC, area under the ROC curve

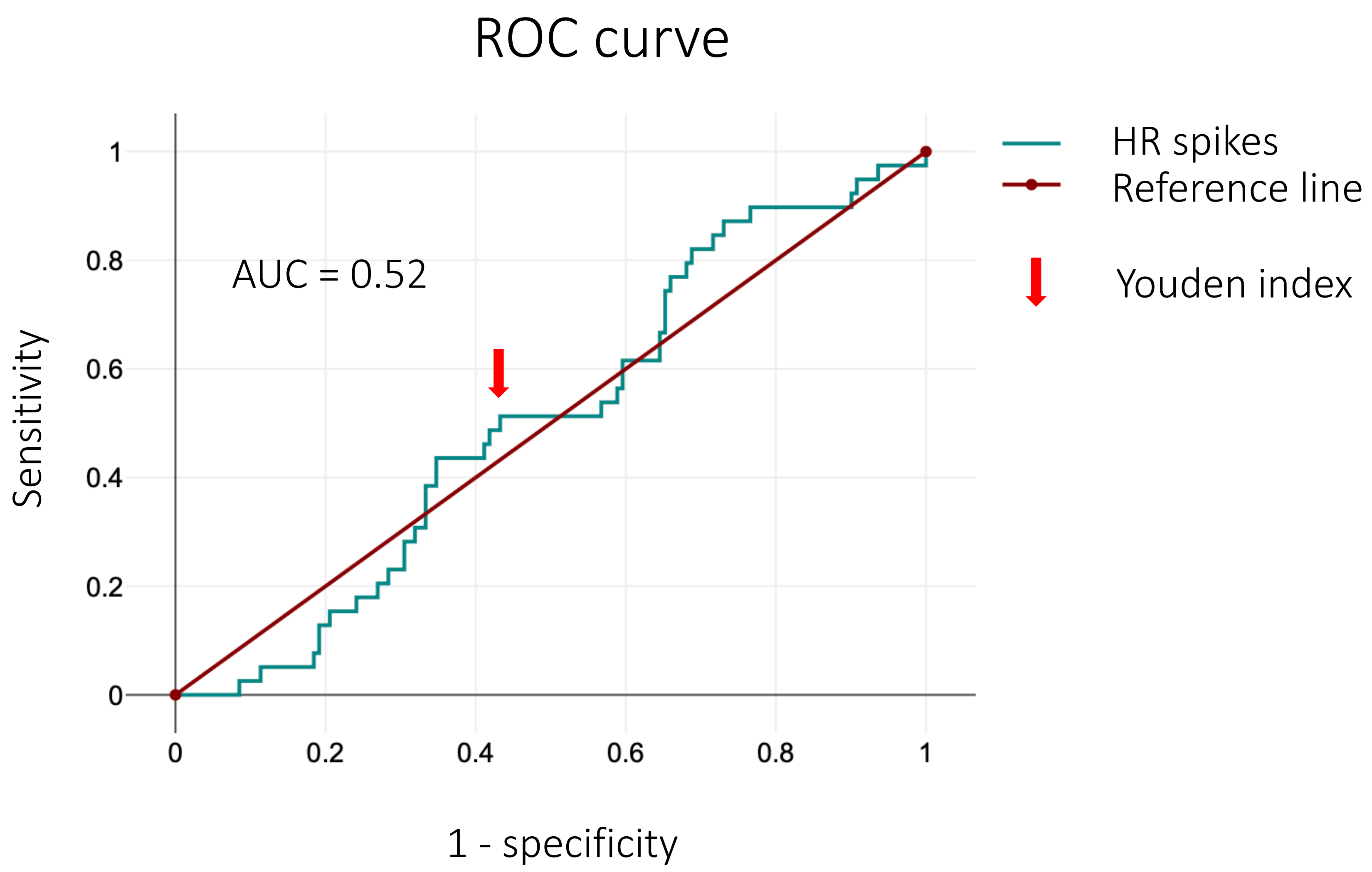

Supplementary Figure 9. Heart rate variability (RMSSD in ms) relevance characteristic curve for postural orthostatic tachycardia syndrome using data from derivation set. AUC, area under the ROC curve
