# Supplementary material for: Screening for postural orthostatic tachycardia syndrome using 24-hour electrocardiogram recording in patients with long coronavirus disease
Source: Heart Rhythm O2. 2025 May 8;6(7):949–55. doi: 10.1016/j.hroo.2025.04.011 (PMC12302153; doi:10.1016/j.hroo.2025.04.011)
Supplement: Supplementary Data [file mmc3.docx]

**Supplementary Table 1.** Contingency tables representing sensitivities and specificities values from the triple analysis (HR spikes, duration of increase in HR after awakening and cardiac dysautonomia) from 24-h ECG recordings.

*The classification accuracy of the test under evaluation is summarized in a 2-by-2 contingency table. Supplementary table 1 shows the contingency table with positive and negative outcomes. Marginal totals are count of subjects for each class: real positives (RP) and real negatives (RN), and predicted positives (PP) and predicted negatives (PN) are determined by the analysis under evaluation, respectively. Letting N represent the total number of subjects, N is equal to RP + RN = PP + PN and the prevalence rate of positive cases is RP/N. True positives (TP) and true negatives (TN) refer to the number of correct classifications for RP and RN, respectively. Conversely, false positives (FP) and false negatives (FN) refer to incorrect classifications for RN and RP, respectively. Sensitivity (Se) is the proportion of RP cases which are correctly predicted positive, whereas specificity (Sp) is the proportion of RN cases which are correctly predicted negative. Positive and negative likelihood ratios (LR+ and LR-) correspond to the ratio of the probability of a positive or negative result in RP subjects to the probability in RN subjects.*

*LR+=Se/1-Sp*

*LR-=1-Se/Sp*

|  | **Real positive (RP)** | **Real negative (RN)** |
| --- | --- | --- |
| **Predicted positive (PP)** | TP | FP |
| **Predictive negative (PN)** | FN | TN |

**Spikes: Long COVID POTS patients vs. healthy subjects**

Se=82.22%

Sp=98%

LR+=41,11

LR-=0,18

|  | **POTS** | **No POTS** |
| --- | --- | --- |
| **Spike ≥ 0.9/h** | 37 | 2 |
| **Spike <0.9/h** | 8 | 98 |

**Spikes: Long COVID POTS patients vs. long COVID no POTS patients**

Se=82.22%

Sp=80%

LR+=4,11

LR-=0,22

|  | **POTS** | **No POTS** |
| --- | --- | --- |
| **Spike ≥ 0.9/h** | 37 | 11 |
| **Spike <0.9/h** | 8 | 44 |

**Spikes + awakenings: Long COVID POTS patients vs. healthy subjects**

Se=86,67%

Sp=99%

LR+=86,67

LR-=0,13

|  | **POTS** | **No POTS** |
| --- | --- | --- |
| ***** | 39 | 1 |
| **#** | 6 | 99 |

**p<0.05* during the first 28 min after awakening compared to healthy subjects

# *p>0.05* during the first 28 min after awakening compared to healthy subjects

**Spikes + awakenings: Long COVID POTS patients vs. long COVID no POTS patients**

Se=86,67%

Sp=90,91%

LR+=9,63

LR-=0,15

|  | **POTS** | **No POTS** |
| --- | --- | --- |
| ***** | 39 | 5 |
| **#** | 6 | 50 |

**p<0.05* during the first 25 min after awakening compared to long COVID no POTS patients

# *p>0.05* during the first 25 min after awakening compared to long COVID no POTS patients

**Spikes + awakenings + HRV: Long COVID POTS patients vs. healthy subjects**

Se=91,11%

Sp=99%

LR+=91,11

LR-=0,09

|  | **POTS** | **No POTS** |
| --- | --- | --- |
| **RMSSD < 35 ms** | 41 | 1 |
| **RMSSD ≥ 35 ms** | 4 | 99 |

**Spikes + awakenings + HRV: Long COVID POTS patients vs. long COVID no POTS patients**

Se=91,11%

Sp=96,36%

LR+=25,03

LR-=0,09

|  | **POTS** | **No POTS** |
| --- | --- | --- |
| **RMSSD < 35 ms** | 41 | 2 |
| **RMSSD** **≥ 35 ms** | 4 | 53 |
