# Supplementary material for: Screening for postural orthostatic tachycardia syndrome using 24-hour electrocardiogram recording in patients with long coronavirus disease
Source: Heart Rhythm O2. 2025 May 8;6(7):949–55. doi: 10.1016/j.hroo.2025.04.011 (PMC12302153; doi:10.1016/j.hroo.2025.04.011)
Supplement: Supplementary Data [file mmc4.docx]

**Supplementary Tables and Figures**

**Supplementary Table 1.** Contingency tables representing sensitivities and specificities values from the triple analysis (HR spikes, duration of increase in RR after awakening and cardiac dysautonomia) from 24-h ECG recordings.

**Supplementary Figure 1.** Detection of heart rate spikes (red arrows) in long COVID POTS patients over the diurnal period of RR intervals derived from 24-h ECG recordings. *Nocturnal period appears as a blue line.*

**Supplementary Figure 2.** Detection of heart rate spikes (red arrows) in long COVID no POTS patients over the diurnal period of RR intervals derived from 24-h ECG recordings. *Nocturnal period appears as a blue line.*

**Supplementary Figure 3.** Detection of heart rate spikes (red arrows) in healthy controls subjects over the diurnal period of RR intervals derived from 24-h ECG recordings. *Nocturnal period appears as a blue line.*

**Supplementary Figure 4**. Analysis of the mean HR (measured each minute, each 2 minutes and each 5 minutes) surrounding the awakening of long COVID POTS patients

**Supplementary Figure 5**. Analysis of the mean HR (measured each minute, each 2 minutes and each 5 minutes) surrounding the awakening of long COVID no POTS patients

**Supplementary Figure 6**. Analysis of the mean HR (measured each minute, each 2 minutes and each 5 minutes) surrounding the awakening of healthy controls subjects

**Supplementary Figure 7**. Heart rate variability analysis over the nocturnal period of RR intervals derived from 24-h ECG recordings

**Supplementary Figure 8.** Heart rate spikes relevance characteristic curve for postural orthostatic tachycardia syndrome using data from derivation set. AUC, area under the ROC curve

**Supplementary Figure 9.** Heart rate variability (RMSSD in ms) relevance characteristic curve for postural orthostatic tachycardia syndrome using data from derivation set. AUC, area under the ROC curve
